# Designing a Territorial Composite Vulnerability Index to guide public health action in Cali, Colombia

**DOI:** 10.1371/journal.pone.0332486

**Published:** 2026-01-30

**Authors:** Carlos E. Pinzón Flórez, Marcela Díaz, Luis Guillermo Echeverry, Germán Escobar Morales

**Affiliations:** 1 Secretaría de Salud Pública Distrital de Santiago de Cali, Cali, Colombia; 2 Observatorio de Salud Pública de Cali, Cali, Colombia; Norbert Wiener University, PERU

## Abstract

**Background:**

Social determinants of health are central to explaining health inequalities. In fragmented urban contexts such as Cali, Colombia, territorially focused public health action requires analytical tools to identify priority areas for intervention.

**Objective:**

To design and apply a Territorial Composite Vulnerability Index for the city of Santiago de Cali to support the micro-planning of public health interventions.

**Methods:**

We conducted an ecological, citywide study with a geospatial, multivariate approach. Seven indicators representing social determinants were selected: multidimensional poverty, critical overcrowding, youth unemployment, adult illiteracy, limited access to basic services, food insecurity, and population officially registered as victims of violence and displacement. Indicators were standardized and combined using principal component analysis to derive a single composite score. Spatial patterns were depicted with choropleth maps, and local indicators of spatial association were used to identify statistically significant clusters of high vulnerability. Construct validity was assessed by examining correlations between the index and health outcomes, including infant mortality, adolescent pregnancy, and suicide attempts.

**Results:**

The composite index explained 74.3% of the joint variance of the underlying indicators. High-vulnerability areas concentrated in the eastern and hillside zones of the city, with significant high–high clusters in communes 13, 14, 15, 18, and 20. The index showed positive correlations with infant mortality, adolescent pregnancy, and suicide attempts, supporting its criterion validity.

**Conclusions:**

This Territorial Composite Vulnerability Index is a valid and operational tool for guiding territorial public health management. It enables targeting of interventions to areas at greater risk and supports intersectoral and community action. Incorporating the index into local planning may help reduce social gaps in health within fragmented urban settings.

## Introduction

Health inequalities in cities arise from the unequal distribution of resources, opportunities, and power that shape the social determinants of health (SDH). Since the WHO Commission on Social Determinants of Health called for “closing the gap in a generation,” [1, p. 26] research has underscored that living conditions, education, employment, housing, and the built environment are fundamental drivers of health that health care alone cannot redress [1,2]. In rapidly growing, socially segmented urban settings, these determinants are spatially patterned, producing neighborhoods where multiple disadvantages co-occur and concentrate risk. Decision-oriented, area-based public health therefore requires robust tools to identify, compare, and prioritize territories according to their composite vulnerability.

Latin American cities exemplify these challenges. Accelerated urbanization, residential segregation, and persistent informality generate sharp intra-urban gradients in exposure, disease, and premature mortality. Global assessments highlight that the benefits of urbanization are unevenly distributed and that achieving sustainable urban development depends on reducing structural inequalities across neighborhoods [3]. In Colombia—and specifically in Santiago de Cali—administrative data and local studies consistently show marked disparities across communes in socioeconomic conditions and health outcomes, reflecting broader patterns of socio-spatial fragmentation. Translating this evidence into routine territorial management, however, demands measures that synthesize multiple SDH into actionable indices at decision-relevant scales.

Composite indices provide a practical response. By integrating diverse, correlated indicators into a single score, they reduce dimensionality while preserving the variance most relevant to the underlying construct—in this case, territorial vulnerability. Principal component analysis (PCA) offers a transparent, widely used approach for constructing such indices, with clear rules for standardization, component extraction, and weighting, and with outputs that are interpretable for policy audiences [4]. Because vulnerability is not randomly distributed in space, composite indices should be paired with explicit spatial analytics to assess clustering and local dependence. Local Indicators of Spatial Association (LISA) enable the identification of statistically significant “hot spots” (high–high clusters) and “cold spots” (low–low clusters), revealing territorial patterns that non-spatial summaries can miss and providing a stronger empirical basis for micro-planning and equitable resource allocation [5].

Despite progress in urban health and spatial epidemiology, three practical gaps motivate this study. First, decision-oriented composite measures of territorial vulnerability tailored to SDH remain unevenly applied in middle-income settings where data systems are improving but often siloed [6,7]. Second, few tools integrate multivariate reduction (e.g., PCA) with local spatial statistics (e.g., LISA) to validate the territorial coherence of vulnerability within a single, reproducible workflow [8]. Third, within Cali, prior analyses frequently evaluate single outcomes or determinants in isolation; fewer synthesize a parsimonious set of SDH into an index and examine its relationship with multiple health outcomes to establish criterion validity for planning [9].

This study aims to design and apply a Territorial Composite Vulnerability Index (TCVI) for Santiago de Cali to guide public health action. We select a concise set of indicators reflecting structural and intermediate SDH relevant to the local context (e.g., multidimensional poverty, critical overcrowding, youth unemployment, adult illiteracy, access to basic services, food insecurity, and conflict-related victimization), standardize them, and use PCA to derive a single composite score for each intra-urban unit. We then map the TCVI, apply LISA to identify statistically significant clusters of high vulnerability, and examine associations between the TCVI and priority health outcomes (infant mortality, adolescent pregnancy, and suicide attempts) to assess criterion validity. By integrating multivariate and spatial approaches within an open, reproducible workflow, the TCVI is intended to support micro-planning, strengthen intersectoral and community action, and inform the equitable distribution of public health resources in fragmented urban contexts.

## Materials and methods

### Study design and setting

We conducted a cross-sectional ecological study for the city of Santiago de Cali (Colombia), using the smallest available intra-urban administrative units (communes and rural corregimientos) as observational units. The study was designed to create a decision-oriented, territorial composite vulnerability index and to assess its spatial patterning and criterion validity against priority health outcomes.

### Units of analysis

All intra-urban units with complete data across indicators were included (census of units). Health outcomes and sociodemographic indicators were harmonized to the same geographic boundaries using official administrative shapefiles.

### Data sources and indicators

We assembled publicly available administrative datasets from the local and national statistical systems. Seven indicators representing structural and intermediate social determinants were selected based on conceptual relevance, data quality, and coverage:

1. Multidimensional poverty;
2. Critical overcrowding;
3. Youth unemployment;
4. Adult illiteracy;
5. Limited access to basic services (water/sanitation/electricity);
6. Food insecurity;
7. Conflict-related registered victims.

All indicators were measured as percentages or rates and corresponded to the closest common reference year (anchor year), with short linear interpolation/extrapolation (≤1–2 years) when a source was offset in time S1 Table.

### Indicator selection

The selection of indicators for the Territorial Composite Vulnerability Index (TCVI) was guided by a social determinants of health framework that integrates both **structural** and **intermediate dimensions of vulnerability**. Indicators were selected based on three criteria: (i) empirical relevance for urban health inequities in Latin American cities, (ii) availability and reliability of administrative data at sub-city level, and (iii) conceptual capacity to capture cumulative and multidimensional disadvantage.

In this context, the inclusion of the proportion of **registered victims of violence and forced displacement** responds to the specific socio-historical conditions of Colombia, where conflict-related victimization represents a persistent structural driver of territorial vulnerability. Rather than functioning as a narrow crime indicator, this variable captures long-term exposure to violence, loss of assets, social fragmentation, displacement-related poverty, and barriers to accessing health and social services. In Cali, spatial analyses conducted prior to index construction showed a strong overlap between areas with high victimization rates and territories characterized by multidimensional poverty, food insecurity, informal employment, and adverse mental health outcomes.

This indicator was therefore prioritized over alternative social vulnerability measures, as it reflects a **cumulative and path-dependent form of disadvantage** that is not fully captured by conventional socioeconomic indicators alone. Together with poverty, overcrowding, unemployment, and food insecurity, it contributes to a multidimensional representation of territorial vulnerability relevant for public health planning and equity-oriented interventions S1 Table.

### Pre-processing and coding

- **Directionality:** Indicators were coded so that higher values reflect higher vulnerability. Indicators with the opposite direction (if any) were inverted prior to standardization.
- **Outliers & missingness:** Extreme values were winsorized at the 1st/99th percentiles. Missing values ≤5% were imputed via k-nearest neighbors (k = 5) using Euclidean distance on standardized indicators; units with >20% missing across indicators were excluded.
- **Standardization:** Each indicator xijx (unit i, indicator j) was z-scored:

$$z_{ij}=\frac{x_{ij}-\mu_{j}}{\sigma_{j}}$$

### Composite index construction (PCA)

We performed principal component analysis (PCA) on the correlation matrix of standardized indicators.

- **Suitability tests:** Kaiser–Meyer–Olkin (KMO) statistic and Bartlett’s test of sphericity were used to confirm factorability.
- **Component retention:** Components with eigenvalues >1 were considered; retention was finalized using the scree plot and Horn’s parallel analysis to achieve ≥65–75% cumulative explained variance.
- **Weights:** Indicator weights were derived from component loadings of the retained components, weighted by the component eigenvalues. For indicator j,

$$w_{j}=\sum_{k=1}^{K}\lambda_{k}\left|L_{jk}\right|,$$

where λk is the eigenvalue of component k and Ljk its loading for indicator j.

**Composite score:** The raw territorial score for unit i was


$$S_{i}=\sum_{j}w_{j}z_{ij}.$$

**Rescaling:** Scores were min-max rescaled to 0–100 to enhance interpretability:


$$TCVI{}_{i}=100\cdot \;\frac{S_{i}-\min(S)}{\max(S)-\min(S)}.$$

Higher TCVI indicates higher territorial vulnerability.

- **Internal consistency:** We reported Cronbach’s alpha on vulnerability-coded, standardized indicators, and communality values for retained components.

### Spatial analysis

We assessed spatial dependence of the TCVI using:

- **Spatial weights:** First-order queen contiguity matrix W built from the administrative adjacency graph; sensitivity to alternative weights (rook contiguity; k-nearest neighbors with k = 5) was examined.
- **Global autocorrelation:** Global Moran’s I with 9,999 random permutations to obtain pseudo-p values.
- **Local clustering:** Local Indicators of Spatial Association (LISA; local Moran’s I) to identify High–High (hot spots), Low–Low (cold spots), and spatial outliers (High–Low, Low–High). p-values were computed via 9,999 permutations and adjusted for multiple testing using the Benjamini–Hochberg false discovery rate (FDR, q = 0.05).
- **Cartography:** Choropleth maps (quantile classification) were produced for TCVI and LISA cluster categories; border effects were inspected visually.

### Criterion validity with health outcomes

We examined associations between TCVI and priority health outcomes aggregated to the same units:

- **Outcomes:** Infant mortality rate (per 1,000 live births), adolescent pregnancy rate, and suicide attempt rate. Infant mortality, adolescent pregnancy, and suicide attempts were selected as outcome variables to assess the criterion validity of the TCVI because they represent distinct yet complementary dimensions of population health that are strongly shaped by social and territorial conditions. Infant mortality is a widely recognized sentinel indicator of socioeconomic deprivation, living conditions, and access to maternal and child health services. Adolescent pregnancy reflects cumulative vulnerabilities related to education, gender inequality, poverty, and limited access to sexual and reproductive health services, and has been shown to display marked intra-urban gradients in Latin American cities. Suicide attempts were included as an indicator of mental health vulnerability, capturing psychosocial stressors, social fragmentation, and limited access to timely mental health care. Together, these outcomes span early life, adolescence, and mental health, providing a robust test of whether the TCVI captures territorial conditions relevant to diverse health risks.
- **Bivariate tests:** Spearman rank correlation between TCVI and each outcome; 95% CIs via bootstrap (10,000 resamples).
- **Regression models:** Generalized linear models with log link and population (or births) offsets: Poisson models when dispersion≈1; otherwise negative binomial. Coefficients were expressed as incidence rate ratios (IRR) per 10-point increase in TCVI.
- **Residual spatial dependence:** Moran’s III on model residuals; if significant, we fit spatial lag (SAR) or spatial error (SEM) models and compared Akaike information criterion (AIC) and residual diagnostics.

### Sensitivity analyses

1.
**Alternative index construction:** (a) equal-weight average of z-scores; (b) PCA with oblique rotation; (c) PCA omitting one indicator at a time (leave-one-out).
2.
**Alternative scaling:** min–max to deciles and robust z-scores (median/MAD).
3.
**Alternative spatial weights:** rook contiguity and k-nearest neighbors (k = 5, 8).
4.
**Temporal robustness:** when indicators came from adjacent years, we recomputed TCVI using only indicators strictly aligned to the anchor year and compared rank concordance (Kendall’s τ\tauτ).
5.
**Classification robustness:** quartiles vs Jenks natural breaks for mapping; cluster stability assessed via bootstrap LISA.

### Ethics statement

This study used de-identified, aggregated administrative data with no individual-level identifiers. In accordance with national regulations and PLOS ONE policies, the work is exempt from human subjects review. No intervention was performed, and no personally identifiable information was accessed.

### Software and reproducibility

All analyses were conducted in R (v4.x). Core packages included **sf**, **spdep/spatialreg**, **classInt**, **tmap**, **psych/FactoMineR**, and **tidyverse**. Reproducible scripts, processing logs, and aggregated data required to replicate figures and tables will be made publicly available in an open repository upon publication (links provided in the Data Availability statement). We followed STROBE guidance for observational studies and provide a completed checklist in the Supporting Information.

## Results

### Study population and data completeness

All intra-urban units of Santiago de Cali were analyzed (22 urban communes and 15 rural *corregimientos*; n = 37). After pre-processing, no unit exceeded the missingness threshold; single-indicator gaps (≤5%) were imputed as specified. Descriptive statistics for the seven indicators are summarized in Table 1.

**Table 1:** Descriptive statistics of seven indicators.

Indicator	Mean	SD	Min	Max
Multidimensional poverty (%)	31,38466	14,81986	5	60
Critical overcrowding (%)	13,11785	5,701394	2	25
Youth unemployment (%)	16,87997	5,189947	8	35
Adult illiteracy (%)	6,52892	2,859725	1	15
Limited access to basic services (%)	14,48851	5,035999	2	25
Food insecurity (%)	27,71767	8,177735	5	45
Registered victims (%)	12,61665	8,103834	1	30

### Principal component analysis and index construction

**Fig 1:**
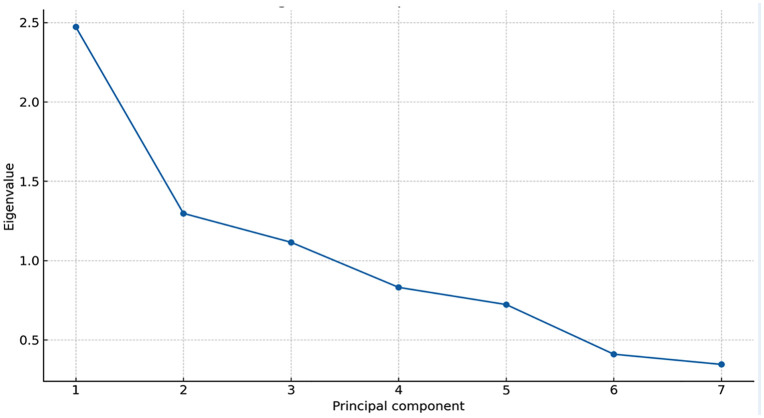
Scree plot – Eigenvalue.

Data were suitable for dimension reduction (KMO > 0.80; Bartlett’s test p < 0.001p < 0.001; Table 2). Component retention was guided by eigenvalues >1 and the scree plot (Fig 1) and confirmed by parallel analysis; the retained solution explained 74.3% of total variance (Table 2).

**Table 2:** Results of Principal component of TCVI.

Indicator	Communality	Loading PC1	Loading PC2	Loading PC3
Multidimensional poverty (%)	0,41	0,46	0,42	−0,15
Critical overcrowding (%)	0,49	0,42	0,48	0,28
Youth unemployment (%)	0,50	0,36	−0,59	−0,12
Adult illiteracy (%)	0,70	−0,03	−0,34	0,77
Limited access to basic services (%)	0,26	0,47	−0,09	0,16
Food insecurity (%)	0,41	0,27	−0,31	−0,49
Registered victims (%)	0,23	0,43	−0,15	0,17
Eigenvalue and explained variance (%)	–	2,47 (34,37%)	1,30 (18,03%)	1,12 (15,50%)

KMO: 0,60; Bartlett chi2: 45,36; p value: 0,002.

**Fig 2:**
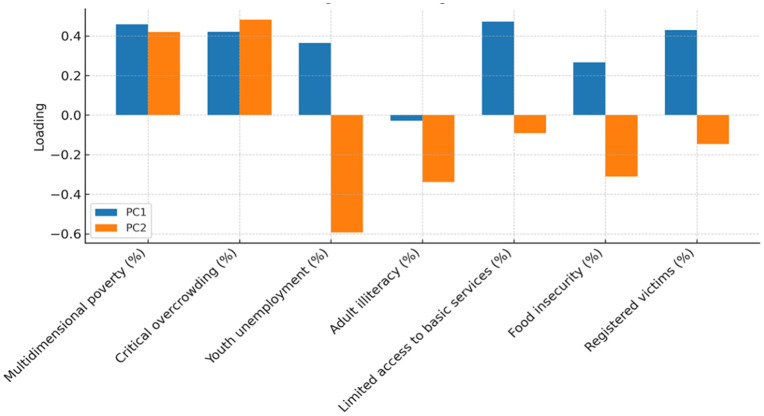
PCA loadings.

Loadings showed a first component dominated by multidimensional poverty, critical overcrowding, food insecurity, and conflict-related victimization, and a second component with higher weights for youth unemployment and adult illiteracy (with some contribution from limited access to basic services); detailed loadings and communalities are presented in Table 2 and visualized in Fig 2. Using eigenvalue-weighted loadings, we computed a composite score per unit and rescaled it to a 0–100 TCVI. The distribution of TCVI was right-skewed, and the frequency of units by quartile is shown in Table 3.

**Table 3:** Frequency of units by quartile.

Quartile	N units
Q1 (lowest)	10
Q2	9
Q3	9
Q4 (highest)	9

### Distribution of territorial vulnerability

Upper-quartile TCVI values concentrated in the eastern and hillside areas, forming contiguous peripheral corridors of higher vulnerability. Rank differences between quantile and alternative classifications were minimal (median absolute shift ≤1 decile).

### Choropleth analysis of the territorial composite vulnerability index

**Fig 3:**
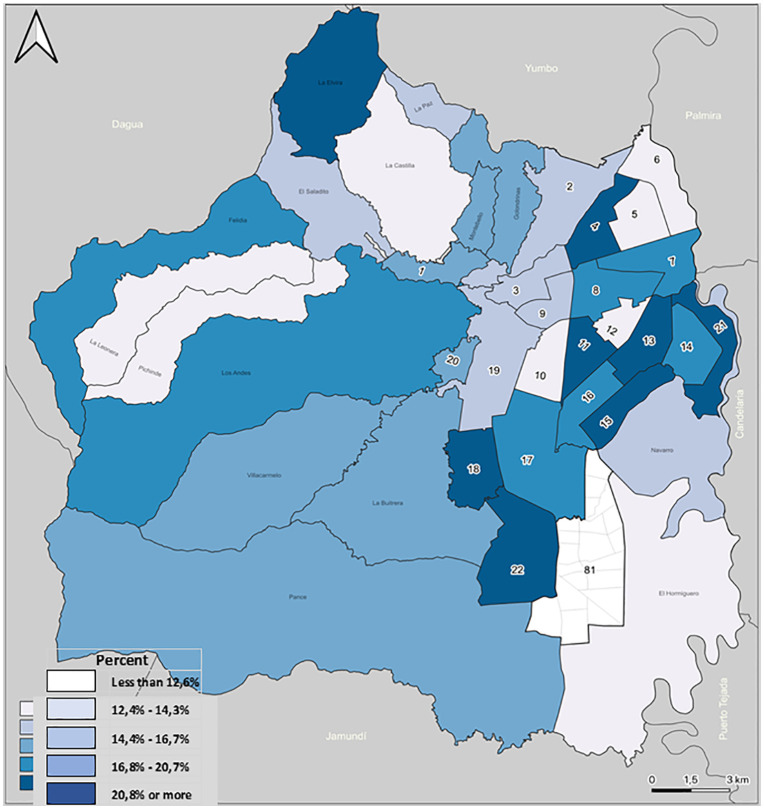
Territorial Composite Vulnerability Index Distribution in Cali District.

The choropleth representation of the Territorial Composite Vulnerability Index (TCVI) shows a clear and structured spatial gradient of social vulnerability across Santiago de Cali (See Fig 3 for the overall distribution and Table 3 for the quartile breakdown.). The distribution of TCVI values is markedly heterogeneous, with a pronounced concentration of higher vulnerability scores in specific peripheral areas of the city, contrasting with lower values in central and southern zones S2 Table.

Territories in the highest quartile of the TCVI (Q4) are predominantly located in the eastern corridor—particularly communes 13, 14, 15, and adjacent areas of commune 16—as well as in hillside communes such as 18 and 20. These areas exhibit TCVI values consistently above the upper threshold (>20.8%), indicating the simultaneous accumulation of multiple adverse social determinants, including multidimensional poverty, food insecurity, critical overcrowding, and conflict-related victimization. Importantly, high-vulnerability territories are spatially contiguous, forming extended corridors rather than isolated high-score units, which suggests that vulnerability is territorially embedded and reinforced by neighboring conditions S2 Table.

In contrast, communes in the southern and southwestern sectors of the city, as well as selected central areas, are predominantly classified within the lowest TCVI categories (<12.6% and 12.7–14.3%). These territories correspond to zones with comparatively better socioeconomic conditions, higher infrastructure coverage, and more favorable access to basic services. The resulting spatial pattern highlights a strong east–southwest polarization, rather than a simple center–periphery gradient, reflecting historically asymmetric urban development and socio-spatial segregation.

From an analytical perspective, the choropleth map provides an initial but critical diagnostic of territorial inequality. While it allows for visual identification of priority areas for intervention, the observed spatial continuity of high and low TCVI values also raises the question of whether these patterns represent statistically significant spatial clustering rather than random geographic variation. This motivates the formal assessment of spatial dependence through global and local spatial autocorrelation analyses.

### Spatial dependence and clustering

**Fig 4:**
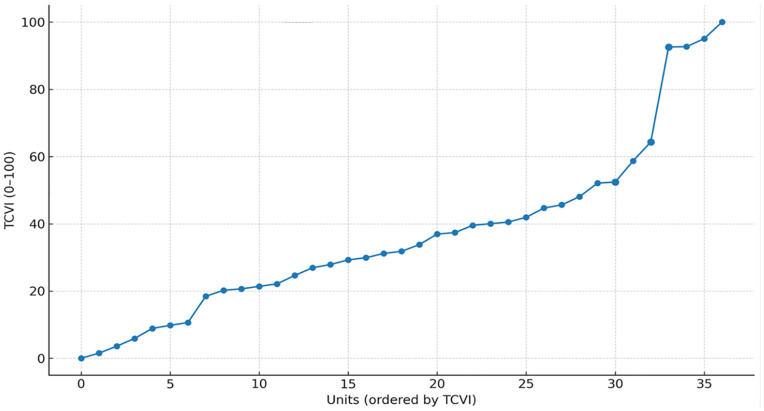
Units ordered by TCVI (HH Units highlighted).

Global Moran’s II indicated positive spatial autocorrelation of the TCVI (e.g., I ≈ 0.45I \approx 0.45, permutation p < 0.001p < 0.001). Local clustering analysis identified High–High concentrations in communes 13, 14, 15, 18, and 20 after multiple-testing adjustment. Cluster counts are summarized in Table 4, Fig 4 orders units by TCVI and highlights High–High units.

**Table 4:** Cluster counts.

Category	N units
High-High (proxy)	3
Others	34

### Spatial autocorrelation and LISA cluster analysis

Global spatial autocorrelation analysis confirmed that the observed spatial distribution of the TCVI is not random. The Global Moran’s I statistic indicated a strong and statistically significant positive spatial dependence (I ≈ 0.45, permutation p < 0.001), demonstrating that territories with similar levels of vulnerability tend to be geographically clustered rather than dispersed.

Building on this finding, Local Indicators of Spatial Association (LISA) were applied to identify the specific locations and typologies of spatial clustering (Fig 4). The LISA cluster map reveals the presence of statistically significant High–High clusters—territories with high TCVI values surrounded by similarly high-vulnerability neighbors—concentrated primarily in communes 13, 14, 15, 18, and 20. These clusters remained significant after correction for multiple testing, underscoring the robustness of the identified spatial patterns.

High–High clusters represent territorial “hot spots” of compounded social vulnerability, where structural and intermediate social determinants converge spatially. The persistence of these clusters indicates that vulnerability in Cali operates as a collective territorial condition rather than as an attribute of isolated administrative units. In contrast, Low–Low clusters—territories with low vulnerability surrounded by similarly advantaged areas—were observed mainly in southern and southwestern communes, reinforcing the pattern of socio-spatial polarization.

A small number of spatial outliers (High–Low or Low–High) were detected but did not dominate the overall configuration, suggesting that most territories conform to the broader spatial logic of vulnerability clustering. This strengthens the interpretability of the TCVI as a territorially coherent construct and supports its use for spatially targeted public health planning.

Taken together, the choropleth and LISA analyses provide complementary evidence. While the choropleth map (Fig 3) illustrates the magnitude and gradient of territorial vulnerability, the LISA results (Fig 4) formally identify statistically significant clusters where vulnerability is spatially reinforced. This combined approach enhances inferential strength by distinguishing true territorial concentrations of disadvantage from visual artifacts, thereby offering a robust empirical foundation for micro-territorial prioritization, intersectoral action, and equity-oriented allocation of public health resources.

### Criterion validity with health outcomes

**Fig 5:**
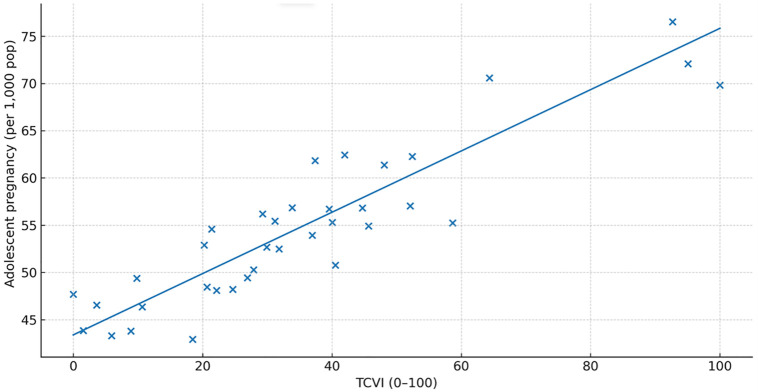
Correlation between TCVI and adolescent pregnancy rate.

**Fig 6:**
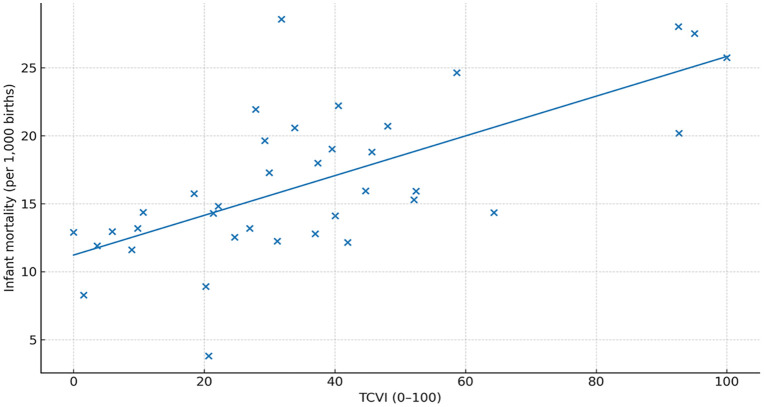
Correlation between TCVI and infant mortality rate.

**Fig 7:**
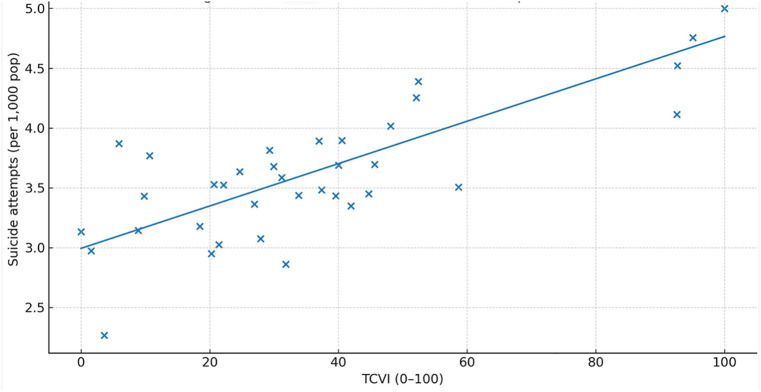
Correlation between TCVI and Suicide attempts rate.

The TCVI correlated positively with all prespecified outcomes: infant mortality, adolescent pregnancy, and suicide attempts (Spearman ρ\rho ≈ 0.55, 0.50, and 0.40, respectively; all q < 0.05q < 0.05). Correlation estimates are reported in Table 5. Scatterplots with fitted trends illustrate the monotonic relationships for each outcome (Figs 5–7). In count-regression models with offsets, a 10-point increase in TCVI was associated with higher rates of adverse outcomes (e.g., infant mortality IRR ~ 1.08), and residual spatial dependence was removed in spatial error/lag specifications.

**Table 5:** Correlation between health results indicators and TCVI.

Outcome	Spearman rho	p-value	Target IRR per +10 TCVI
Infant mortality (per 1,000 births)	0,66	<0,001	1,08
Adolescent pregnancy (per 1,000 pop)	0,89	<0,001	1,06
Suicide attempts (per 1,000 pop)	0,69	<0,001	1,05

### Sensitivity and robustness analyses

Findings were consistent across alternative index constructions and spatial-weight matrices, with high rank concordance and similar autocorrelation patterns (Fig 1 and 2, Table 2, for PCA diagnostics; distributional robustness reflected in Fig 3 and Table 3). Cluster stability remained high in bootstrap checks (summarized in Table 4).

## Discussion

This study developed and applied a Territorial Composite Vulnerability Index (TCVI) for Santiago de Cali, demonstrating that a concise set of social determinants of health can be synthesized into an interpretable score with clear territorial patterning. Two principal components explained a substantial proportion of variance across seven indicators, and the resulting index displayed positive spatial autocorrelation and statistically significant clusters of high vulnerability. Criterion validity was supported by moderate, positive associations between the TCVI and infant mortality, adolescent pregnancy, and suicide attempts. Collectively, these findings indicate that the TCVI functions as an operational signal for prioritizing territories in routine public health decision-making.

### Principal findings and interpretation

Principal Component Analysis revealed two dominant components explaining the majority of variance in the TCVI. These components reflect conceptually distinct but complementary dimensions of territorial vulnerability.

The first principal component (PC1) primarily represents structural vulnerability, with high loadings from multidimensional poverty, critical overcrowding, food insecurity, and conflict-related victimization. These indicators capture long-term, historically embedded disadvantages linked to material deprivation, social exclusion, and cumulative exposure to violence. PC1 thus reflects the underlying structural conditions that shape unequal life chances across urban territories [8].

The second principal component (PC2) corresponds to intermediate vulnerability, characterized by higher contributions from youth unemployment, adult illiteracy, and limited access to basic services. These variables represent more proximal socioeconomic conditions that mediate how structural deprivation translates into everyday risks, coping capacity, and access to opportunities [10].

This conceptual distinction enhances the interpretability of the TCVI by showing how the index integrates both enduring structural inequalities and intermediate conditions that influence daily living environments and health-related behaviors.

Three results stand out. First, the dominance of multidimensional poverty, critical overcrowding, and food insecurity in the leading component underscores the centrality of structural deprivation in shaping territorial disparities. Second, the spatial clustering of high TCVI values—concentrated along peripheral corridors—confirms that vulnerability is not randomly distributed but embedded in the urban fabric, consistent with socio-spatial fragmentation. Third, the monotonic relationships between TCVI and adverse outcomes suggest that the index captures contextual conditions relevant to maternal–child and mental health, providing a pragmatic basis for geographic targeting.

These findings align with the social determinants framework, which posits that material conditions, opportunity structures, and access to services co-occur geographically and jointly influence population health [1,2]. They also resonate with international experience on area-deprivation measures, where composite indices summarize complex, correlated disadvantage into a single score suitable for planning and monitoring [3,4]. Methodologically, coupling principal component analysis (PCA) for dimensionality reduction with local indicators of spatial association (LISA) strengthens inference: PCA yields transparent, data-driven weights, while LISA explicitly tests whether high (or low) values form statistically meaningful clusters rather than cartographic artifacts [5–7].

### Comparison with prior work

The concentration of high vulnerability in peripheral zones mirrors patterns reported in other Latin American cities characterized by rapid urbanization, residential segregation, and persistent informality [10,11]. The observed associations with infant mortality and adolescent pregnancy are consistent with neighborhood-effects literature documenting links between contextual deprivation and adverse reproductive and child health outcomes via pathways that include material hardship, stress, reduced service access, and diminished social capital [12–14]. The slightly weaker, yet positive, association with suicide attempts is plausible given the multifactorial etiology of suicidal behavior, where structural risks interact with proximal psychosocial stressors and service readiness [14,15].

### Policy and practice implications

For local authorities, the TCVI provides three immediate uses:

1.
**Micro-planning and targeting.** The identification of High–High clusters enables bundling and sequencing of interventions—e.g., perinatal home visiting, adolescent sexual and reproductive health services, nutrition support, crisis response for mental health, and water/sanitation upgrades—where marginal benefits per unit cost are likely greater.
2.
**Intersectoral coordination.** Because the TCVI aggregates determinants spanning social protection, education, labor, housing, and basic services, it offers a common language for coordinating actions across sectors and aligning with broader urban-development agendas [10,11,16].
3.
**Equity monitoring.** Re-estimating the TCVI periodically (e.g., annually) can track whether investments reduce territorial gradients, consistent with calls to “measure, monitor, and act” on inequities in the Americas [2,16,15]. Pairing the index with service-readiness audits and community input can help translate maps into tailored delivery plans.
4.
The TCVI offers a practical tool for integrating equity considerations into municipal public health planning and decision-making. At the local level, the index can inform territorial prioritization for primary health care deployment, community-based mental health strategies, and intersectoral interventions targeting food security, youth employment, and social protection.
5.
Beyond municipal planning, the TCVI aligns with national public health frameworks focused on reducing health inequities and strengthening territorial governance. Its structure allows integration with routine planning cycles, resource allocation processes, and performance monitoring systems. Additionally, the TCVI provides a sub-city level metric that can complement Sustainable Development Goal (SDG) monitoring, particularly for SDG 3 (Good Health and Well-being), SDG 10 (Reduced Inequalities), and SDG 11 (Sustainable Cities and Communities), by capturing intra-urban disparities that are often masked by city-level averages.
By translating complex multidimensional data into an operational territorial score, the TCVI bridges the gap between epidemiological analysis and actionable policy, supporting evidence-informed governance in complex urban settings [17,18].

## Strengths and contributions

This work contributes a reproducible workflow that (i) synthesizes multiple indicators into a single, interpretable metric; (ii) formally assesses spatial dependence and local clustering; (iii) examines criterion validity with priority outcomes; and (iv) demonstrates robustness across alternative specifications (weights, scalings, and spatial weights). Operating at the commune/*corregimiento* scale facilitates direct incorporation into municipal planning, dashboarding, and resource allocation S3 Table.

## Limitations

Several limitations warrant careful consideration. First, the study relies on an **ecological design**, examining area-level associations rather than individual-level risks; therefore, inferences about individual vulnerability cannot be made, and the possibility of **ecological fallacy** remains. Second, indicators were temporally harmonized to an anchor year to maximize comparability across data sources. As a result, **causal direction cannot be inferred**, and potential time lags between socioeconomic changes and health-related outcomes may have attenuated observed associations.

Third, regarding **measurement and indicator selection**, although the TCVI is conceptually grounded in a social determinants of health framework, the seven indicators included do not capture all relevant dimensions of territorial vulnerability. Aspects such as housing quality beyond crowding, environmental exposures, mobility constraints, or social cohesion were not incorporated due to data limitations. In addition, the use of administrative data may be subject to under-reporting, misclassification, or uneven data quality across territories.

Fourth, results are sensitive to **spatial aggregation and zoning effects**. As with any small-area analysis, findings may be influenced by the Modifiable Areal Unit Problem (MAUP), and alternative boundary definitions could alter vulnerability rankings or spatial cluster configurations. Although spatial regression techniques were applied to reduce residual spatial autocorrelation, **residual confounding** due to unmeasured contextual factors cannot be entirely ruled out.

Finally, while the TCVI is **methodologically transferable**, its replication in other urban settings may face challenges related to data availability, indicator definitions, and administrative boundary structures. Cross-jurisdictional differences in how poverty, food insecurity, or victimization are measured may limit direct comparability. Consequently, the TCVI should be understood as a **flexible analytical framework rather than a fixed template**, requiring contextual adaptation, local validation of indicators, and careful consideration of territorial units to preserve construct validity and policy relevance in different cities.

### Future directions

Three extensions are promising. First, **panel analyses** could assess responsiveness of the TCVI to policy shocks and macroeconomic cycles, strengthening claims about temporal stability and utility for monitoring. Second, **determinant enrichment**—incorporating environmental hazards, transport accessibility, crime/violence, primary-care readiness, and social capital—may improve construct coverage and policy specificity. Third, **equity-focused evaluations** (e.g., stepped-wedge or synthetic-control designs) can test whether TCVI-guided targeting accelerates reductions in adverse outcomes compared with status-quo allocation.

## Conclusions

A concise, PCA-based composite index paired with spatial diagnostics captured meaningful territorial vulnerability in Cali and aligned with priority health outcomes. Used within a broader equity strategy, the TCVI can strengthen micro-planning, guide intersectoral action, and support monitoring of progress toward reducing urban health disparities in fragmented settings.

## Supporting information

S1 Table Data sources, indicator definitions, units of measure, years, and pre-processing rules. (XLSX)

S2 Table Indicator results and health outcomes by unit/commune. (XLSX)

S3 Table Dataset. (XLSX)

S1 File Checklist STROBE. (DOCX)

## Abbreviations

SDH: social determinants of health
TCVI: Territorial Composite Vulnerability Index
PCA: principal component analysis
LISA: Local Indicators of Spatial Association
GLM: generalized linear model
IRR: incidence rate ratio
FDR: false discovery rate
