# Supplementary material for: Designing a Territorial Composite Vulnerability Index to guide public health action in Cali, Colombia
Source: PLoS One. 2026 Jan 30;21(1):e0332486. doi: 10.1371/journal.pone.0332486 (PMC12858007; doi:10.1371/journal.pone.0332486)
Supplement: S1 File — (DOCX) [file pone.0332486.s004.docx]

S1 File. Checklist: STROBE

Manuscript: Territorial Composite Vulnerability Index (TCVI) for Santiago de Cali

| **Item No.** | **Item** | **Recommendation (abridged)** | **Where reported** | **Section/Figure/Table refs** | **Notes** |
| --- | --- | --- | --- | --- | --- |
| 1a | Title | Indicate the study design in the title or abstract. | Title; Abstract | Title page; Abstract | Design stated as cross-sectional ecological study. |
| 1b | Abstract | Provide an informative and balanced summary of what was done and found. | Abstract | Abstract | Structured abstract per PLOS ONE guidance. |
| 2 | Background/rationale | Explain the scientific background and rationale for the investigation. | Introduction | Introduction ¶1–3 |  |
| 3 | Objectives | State specific objectives, including any prespecified hypotheses. | Introduction | Introduction final ¶ | Objectives specify TCVI design, mapping, LISA, and criterion validity. |
| 4 | Study design | Present key elements of study design early in the paper. | Materials and methods | Study design and setting | Ecological, cross-sectional; decision-oriented index. |
| 5 | Setting | Describe the setting, locations and relevant dates. | Materials and methods | Study design and setting; Units of analysis | Santiago de Cali; communes and corregimientos; anchor year. |
| 6 | Participants | Give eligibility criteria and the sources and methods of selection. | Materials and methods; Results | Units of analysis; Study population and data completeness | Census of all units with complete data (n=37). |
| 7 | Variables | Clearly define all outcomes, exposures, predictors, confounders. | Materials and methods | Data sources and indicators | Seven SDH indicators; outcomes: IMR, adolescent pregnancy, suicide attempts. |
| 8 | Data sources/measurement | For each variable, give sources of data and details of assessment. | Materials and methods | Data sources; Pre-processing; Composite construction; Spatial analysis | Administrative datasets; standardization; winsorization; imputation. |
| 9 | Bias | Describe any efforts to address potential sources of bias. | Materials and methods; Discussion | Pre-processing; Sensitivity analyses; Limitations | Outlier handling, missingness, robustness checks; ecological caveats. |
| 10 | Study size | Explain how the study size was arrived at. | Materials and methods; Results | Units of analysis; Study population | Complete coverage of all administrative units. |
| 11 | Quantitative variables | Explain how quantitative variables were handled in the analyses. | Materials and methods | Pre-processing and coding; Rescaling; PCA weighting | Z-scoring, min–max rescaling, eigenvalue-weighted loadings. |
| 12 | Statistical methods | Describe all statistical methods and sensitivity analyses. | Materials and methods | Composite index construction; Spatial analysis; Criterion validity; Sensitivity analyses | PCA, Moran’s I, LISA, GLMs with offsets, spatial lag/error models, bootstrap CIs, FDR. |
| 13 | Participants | Report numbers of individuals at each stage. | Results | Study population and data completeness | All units included; imputation ≤5% per indicator. |
| 14 | Descriptive data | Give characteristics of study participants (and information on exposures). | Results; Supporting Information | Table 1; Table 3; Fig 3; S1 Table | Indicator distributions and TCVI quartiles. |
| 15 | Outcome data | Report numbers of outcome events or summary measures. | Results; Supporting Information | Figs 4–6; Table 5; Supplement | Rates for IMR, adolescent pregnancy, suicide attempts. |
| 16 | Main results | Provide unadjusted and, if applicable, adjusted estimates and precision. | Results | Table 2, 2b; Figs 1–2; Table 5; text | Variance explained; loadings; correlations/IRRs with CIs. |
| 17 | Other analyses | Report other analyses done—e.g., sensitivity analyses. | Results | Sensitivity and robustness analyses | Alternative weights/scalings; spatial weights; bootstrap LISA. |
| 18 | Key results | Summarize key results with reference to study objectives. | Discussion | Principal findings |  |
| 19 | Limitations | Discuss limitations of the study. | Discussion | Limitations | Ecological fallacy, timing, measurement, MAUP, residual confounding. |
| 20 | Interpretation | Provide a cautious overall interpretation of results. | Discussion | Interpretation; Policy implications | Context with prior literature; plausible mechanisms. |
| 21 | Generalisability | Discuss the generalisability (external validity) of the study results. | Discussion | Policy and practice implications | Applicability to other Colombian/Latin American cities. |
| 22 | Funding | Give the source of funding and the role of the funders. | Final sections | Funding | No specific grant / or insert funder details. |
| A1 | Ethics | Ethics approval and consent (if applicable). | Final sections | Ethics Statement | Exempt; aggregated, de-identified data. |
| A2 | Data availability | Data-sharing statement with repository/DOI. | Final sections | Data Availability | Open repository with code and derived data. |

Note: This checklist is adapted from the STROBE statement (cross-sectional). References to “Where reported” and “Section/Figure/Table refs” correspond to the sections and materials in this manuscript.
